# Tailoring Stroke Counseling for Risk Reduction Intervention to African American Men

**DOI:** 10.1093/geroni/igab046.1630

**Published:** 2021-12-17

**Authors:** Dawn Aycock, J Taylor Harden, Laura Salazar, Gayenell Magwood, Patricia Clark

**Affiliations:** 1 Georgia State University, Atlanta, Georgia, United States; 2 University of Texas at Austin, Austin, Texas, United States; 3 Medical University of South Carolina, Charleston, South Carolina, United States

## Abstract

Early life course achievement and maintenance of ideal cardiovascular health is associated with reduced risk of developing stroke later in life. The Stroke Counseling for Risk Reduction (SCORRE) intervention is an age-and-culturally relevant intervention originally designed to correct inaccurate stroke risk perceptions and improve lifestyle behaviors to reduce stroke risk in AAs age 20-35. In a study testing SCORRE, fewer men participated, but most were not at a stage of readiness for behavior change; many did not think they were at risk despite averaging three modifiable risk factors, and while improvements in outcomes were observed in women they were not in men. These differences led to tailoring SCORRE to young AA men. The methods for tailoring SCORRE and resulting strategies for attracting, engaging, and empowering them towards stroke risk reduction, including hypotheses concerning food supply, housing, economic and social relationships, education, and mental health care will be raised for discussion.

